# Bomb-pulse ^14^C analysis combined with ^13^C and ^15^N measurements in blood serum from residents of Malmö, Sweden

**DOI:** 10.1007/s00411-013-0458-9

**Published:** 2013-01-29

**Authors:** Elisavet Georgiadou, Kristina Eriksson Stenström, Cintia Bertacchi Uvo, Peter Nilsson, Göran Skog, Sören Mattsson

**Affiliations:** 1 Division of Nuclear Physics, Department of Physics, Lund University, Box 118, 221 00 Lund, Sweden; 2 Water Resources Engineering, Lund University, Box 118, 221 00 Lund, Sweden; 3 Department of Clinical Sciences Malmö, Skåne University Hospital, Lund University, 205 02 Malmö, Sweden; 4 Radiocarbon Dating Laboratory, Lund University, Box 118, 221 00 Lund, Sweden; 5 Department of Clinical Sciences, Medical Radiation Physics, Skåne University Hospital, Lund University, 205 02 Malmö, Sweden

**Keywords:** Serum, Bomb-pulse dating, Stable isotopes

## Abstract

The ^14^C content of 60 human blood serum samples from residents of Malmö (Sweden) in 1978, obtained from a biobank, has been measured to estimate the accuracy of ^14^C bomb-pulse dating. The difference between the date estimated using the Calibomb software and sampling date varied between −3 ± 0.4 and +0.2 ± 0.5 years. The average age deviation of all samples was −1.5 ± 0.7 years, with the delay between production and consumption of foodstuffs being probably the dominating cause. The potential influence of food habits on the ^14^C date has been evaluated using stable isotope δ^13^C and δ^15^N analysis and information about the dietary habits of the investigated individuals. Although the group consisting of lacto-ovo vegetarians and vegans (pooled group) was not completely separated from the omnivores in a stable isotopic trophic level diagram, this analysis proved to add valuable information on probable dietary habits. The age deviation of the sampling date from the respective Calibomb date was found strongly correlated with the δ^13^C values, probably due to influence from marine diet components. For the omnivore individuals, there were indications of seasonal effects on δ^13^C and the age deviation. No significant correlation was found between the age deviation and the δ^15^N values of any dietary group. No influence of sex or year of birth was found on neither the ^14^C nor the δ^13^C and δ^15^N values of the serum samples. The data were also divided into two groups (*omnivores* and *pooled group*), based on the level of δ^15^N in the samples. The consumption of high δ^15^N-valued fish and birds can be responsible for this clustering.

## Introduction

### ^14^C in the environment

^14^C is naturally produced in the upper atmosphere, when nitrogen atoms of the air react with neutrons produced by cosmic rays. Nuclear weapons testing in the atmosphere in the 1950s and early 1960s produced large amounts of ^14^C, which gave rise to the so-called ^*14*^ *C bomb pulse*. In 1963, the specific activity of ^14^C in atmospheric CO_2_ had almost doubled compared to the time before the tests. After the Limited Test Ban Treaty in 1963, the ^14^C concentration in the atmosphere decreased rapidly mainly due to diffusion of ^14^CO_2_ into the oceans (where it is converted into carbonic acid and bicarbonate). Since the ^14^C produced in the atmosphere was mainly bound in carbon dioxide, the bomb pulse has been recorded in all parts of the biosphere. Thus, terrestrially living organisms from the second half of the twentieth century (see e.g. Harkness and Walton 1969, 1972; Wild et al. 2000; Goodsite et al. 2001) can be dated, in some cases with a precision down to ±1 year or even better. The technique has also been widely applied to date human cells and tissues, for example, to obtain information on the formation of new cells in the human body and to date the progress of various states of illness (Broecker et al. 1959; Libby et al. 1964; Harkness and Walton 1969; Nydal et al. 1971; Druffel and Mok 1983; Spalding et al. 2005a, b, 2008; Lynnerup et al. 2008; Gonçalves et al. 2010).

The question of whether clean-air atmospheric ^14^CO_2_ data are representative for samples used for bomb-pulse dating of human materials has been raised previously (Stenström et al. 2010; Georgiadou and Stenström 2010). Clean-air ^14^C data sets are available for different geographical zones (see e.g. Hua and Barbetti 2004; Reimer et al. 2004; and Levin et al. 2008). However, bomb-pulse dating of human tissue can be influenced by inter-individual variations in the ^14^C-specific activity of the diet. Vegetation in the vicinity of nuclear installations such as nuclear power plants reprocessing facilities for spent nuclear fuels and laboratories using ^14^C-labeled material often exhibit elevated levels of ^14^C compared to clean air (Stenström et al. 2010), and thus foodstuffs produced in these areas are expected to contain excess ^14^C. The opposite effect can be seen in heavily industrialized regions, where fossil fuel emissions have lowered the specific activity of ^14^C in foodstuffs (Broecker et al. 1959). It is also important to take the delayed introduction of ^14^C into the human tissues compared to the atmosphere into consideration. Several studies (Nydal et al. 1971; Broecker et al. 1959; Libby et al. 1964; Harkness and Walton 1969) have shown that ^14^C in human organs and fluids lags several months behind the ^14^C of the atmosphere. As pointed out by Nydal et al. (1971), the delay time from growth of vegetables and their consumption by humans may vary. A varied diet also contains meat products, which generally have longer delay time of ^14^C from the atmosphere to human consumption than vegetal foods (Broecker et al. 1959).

Another factor determining the specific activity of ^14^C in the diet is the reservoir from which the diet originates. During the bomb pulse, foodstuffs from terrestrial sources had significantly higher ^14^C content than those from marine sources due to the delay in bomb ^14^C entering the oceans (Scourse et al. 2012, Gordon and Harkness 1992). Thus, the composition and origin of the diet may affect bomb-pulse dating of human tissues on an individual basis. (For human blood, Broecker et al. (1959) estimated that the mean residence time of carbon in blood is <6 months). Internally deposited ^14^C-labeled compounds have shown residence times varying from a few hours to several months in human volunteers (Nydal et al. 1971; Broecker et al. 1959; Harkness and Walton 1969). The generic biokinetic model introduced in ICRP Publication 30 (International Commission on Radiological Protection, 1981) assumes that internally deposited carbon is uniformly distributed throughout the body and is removed with a half-time of 40 days, which means a mean residence time of about 2 months.

### Stable isotope analysis

**Fig. 1:**
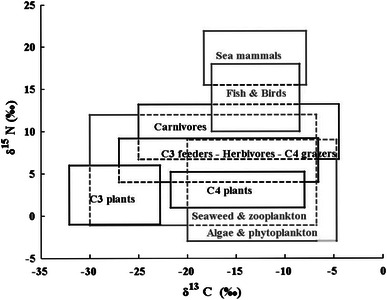
Generalized isotopic trophic level diagram for terrestrial and marine food webs. (Adapted from O’Connell 1996, with permission)

Isotope fractionation is a process that occurs during chemical reactions and physical processes due to the difference in mass between the isotopes. It represents a partial separation of the different isotopes and results in enrichment or depletion of one isotope relative to another (Gillespie 1984; Kutschera 2005; Schoeller 1999). In the carbon cycle, isotope fractionation takes place when carbon is transferred from one part of the ecosystem to another. When carbon from atmospheric CO_2_ is incorporated into vegetation during photosynthesis, ^12^C is absorbed relatively more than ^13^C and ^13^C is absorbed more than ^14^C. There are three main photosynthetic pathways. Most plants, including flowering plants, trees, most of the temperate zone grasses, wheat, potatoes, follow the so-called C_3_ pathway, in which the intermediate product is a molecule containing three carbon atoms (phosphoglyceric acid). The C_4_ photosynthetic pathway, on the other hand, followed, for example, by corn and sugar cane, is leading to a four-carbon molecule (oxaloacetate) as the intermediate product. Finally, the Crassulacean acid metabolism (CAM) photosynthetic pathway is followed, for example, by tropical plants as pineapples and various cacti, only a few of which are included in herbivore and human diet (Farquhar et al. 1989; Craig 1954; van Norman and Brown 1952; Pollard and Heron 2008). Marine plants follow a pathway similar to the C_3_ pathway, but their carbon originates from dissolved marine bicarbonates, whose isotope ratios differ from the atmospheric CO_2_. So enrichment on ^13^C in the marine plants compared to the terrestrial ones is observed (Craig 1954). The isotope fractionation of carbon is expressed as δ^13^C, which is defined as the relative deviation of the ^13^C/^12^C ratio of the sample compared to that of a standard material (Fry and Sherr 1984; Gillespie 1984). Each of the photosynthetic pathways described above creates different isotopic fractionation (Chisholm 1989). The average δ^13^C values of the C_3_ plants is about −28.1 ‰, while the C_4_ pathway results in δ^13^C values of about −13.5 ‰ (Schoeller et al. 1986 and references therein).^1^ δ^13^C values of C4 plants and fishes considerably overlap as one can see in Fig. 1. So, another parameter, as the δ^15^N, is needed to specify more precisely the food source (O’Connell 1996; Schoeninger and DeNiro 1984).

In analogy with the isotope fractionation of carbon, δ^15^N expresses the isotope fractionation of the stable nitrogen ^15^N compared to the most common nitrogen isotope ^14^N (Robinson 2001). Minagawa and Gawa (1984) and Schoeller (1999) both suggested that there is a gradual enrichment of δ^15^N along the food chain. As clearly shown by Minagawa (1992), both δ^13^C and δ^15^N differ between the major food groups of a diet; δ^15^N for food originating from a marine environment being considerably higher than for food from other sources. This can also be deduced from the isotopic trophic level diagram (δ^13^C vs. δ^15^N values) of O’Connell (1996), presented in Fig. 1 and the study of the North American food web by Schoeller et al. (1986). In the human body, δ^13^C values are in general determined by the type of food ingested during the previous several weeks and months (Gearing 1991). Thus, δ^13^C is a good indicator of the average diet, although there are a number of sources of variability which must be taken into account (Gearing 1991). Isotope fractionation between ingested food and human tissue is complex (Schoeller 1999) and depends, for example, on differences in the metabolic breakdown of the various ingested food components. Tieszen et al. (1983) and DeNiro and Epstein (1978) believe that δ^13^C dietary analysis should not be based on the analysis of a single type of tissue. However, Schoeller et al. (1986) estimated that the differences in δ^13^C between diet and the components of the blood are small: the tissue–diet difference was about +0.1 ‰ for plasma protein, −0.1 ‰ for plasma carbohydrates and +0.9 ‰ for plasma lipid. As far as δ^15^N is concerned, DeNiro and Epstein (1981) and references therein proved that the δ^15^N values in animals (whole body or individual tissues and biochemical components) represent the δ^15^N values of their respective diets satisfactorily. Vanderklift and Ponsard (2003) reported that the δ^15^N values of human tissues are commonly higher than the respective values of their diets, depending on the sort of diet and the tissue analyzed. δ^15^N values can, however, be considered quite similar among different organisms. Schoeller et al. (1986) showed that the difference in δ^15^N between plasma protein and diet is about 4.2 ‰. Since the stable ^13^C and ^15^N values of animals and humans (Schoeller et al. 1986) reflect the different dietary sources quite well, with small time lag, they can be used to provide information on the dietary habits.

In a previous study, Georgiadou and Stenström (2010) modeled the potential effect of the marine component of the diet on bomb-pulse dating of human tissue. It was found that food originating from the marine environment could lead to a few years’ delay in radiocarbon dating compared to food of terrestrial origin. In the current study, accelerator mass spectrometry (AMS) was used to date 60 human serum samples collected in 1978 from residents of the city of Malmö in southern Sweden. One purpose was to assess the magnitude of inter-individual variations in human ^14^C data depending on dietary habits, in order to estimate the precision and accuracy of bomb-pulse dating in human tissues. The possible influence of age and sex was also studied. It was also investigated if analysis of the isotope fractionation of stable carbon and nitrogen isotopes (δ^13^C and δ^15^N) using isotope ratio mass spectrometry (IRMS) could provide additional information about the diet to facilitate a correction of the age determined by radiocarbon dating.

## Materials and methods

### Subjects and samples

For the current study, the available human material was blood serum, which is whole blood minus both the blood cells and the clotting factors. Sixty human serum samples (25 μl each) were obtained from the biobank of the Malmö Prevention Project (MPP) in Malmö, Sweden (following ethical approval by the Regional Ethics Committee in Lund, official record no. 85/2004). All individuals were residents of the city of Malmö at the time of sampling. Each subject submitting a sample to the biobank filled an extensive questionnaire, including information such as sex, sampling date and year of birth as well as subjects’ dietary habits. Based on dietary habits, the subjects included in the present study were divided into three groups: (a) *vegans* (consuming only vegetables), (b) *lacto*-*ovo* vegetarians (consuming vegetables, milk products and eggs, but not meat and fish) and (c) *omnivores* (having a varied diet also containing meat and fish). No information was available about the percentage of meat and fish in the diet.

Our original intention was to investigate serum samples from 30 men and 30 women in three different age groups (10 men and 10 women in each) with at least three vegans of both sexes in each age group. Furthermore, we wanted to analyze samples collected during the same year, preferably in the 1970s. One reason for this choice of time is that the declining ^14^C bomb-pulse curve was still steep at the time, which makes the analysis more sensitive than when choosing a later flatter part of the curve. Another reason is that in the 1970s, the diet of the subjects most likely originated from the time after 1963, thus avoiding the influence of the increase in the bomb-pulse curve that occurred before 1963. However, the number of serum samples available from the 1970s at the Malmö biobank was limited; so all these requirements could not be fulfilled. Female subjects and female vegans, in particular, were underrepresented. Therefore, subjects categorized as lacto-ovo vegetarians were also included in the study. The best choice of sampling year was 1978, and the years of birth for the three age groups that best fulfilled the requirements were 1931, 1938 and 1949 (ages of 47, 40 and 29, respectively, at the time of sampling). The characteristics of the subjects are listed in Table 1.

**Table 1:** Characteristics of the subjects included in the study. Year of sampling: 1978. N means number of subjects

Year of birth	Age at sampling (*y*)	Sex	Omnivores (*N*)	Vegans (*N*)	Lacto-ovo vegetarians (*N*)	Total
1931	47	Female	10	0	0	10
1931	47	Male	4	3	3	10
1938	40	Female	10	0	0	10
1938	40	Male	8	1	1	10
1949	29	Female	4	2	4	10
1949	29	Male	10	0	0	10
Total			46	6	8	60

### Sample preparation and analysis

The serum samples obtained from the Malmö biobank were kept in a freezer at −25 °C in small plastic tubes until analysis. The serum samples were thawed for 2 h at room temperature prior to sample preparation for AMS (^14^C) and IRMS (δ^13^C and δ^15^N) analysis.

#### Bomb-pulse dating using ^14^C measurements

Prior to ^14^C analysis, 10 μl of each serum sample was transferred from the plastic tubes into separate small quartz tubes (6.4 mm (diam.) × 45 mm), which had been cleaned at 950 °C in air for 1 h. The small quartz tubes containing the serum samples were then placed in an oven at 80 °C for 1 h to dry the samples. A total of 100 mg of pre-cleaned CuO needed for the combustion was then added to the samples, and the small quartz tubes were inserted into longer (9.5 mm (diam.) × 180 mm) quartz tubes. The quartz tubes were air-evacuated and sealed using a hydrogen/oxygen torch, to capture the CO_2_ produced by the combustion that followed. The tubes were afterward placed in an oven for 2 h to reach a temperature of 950 °C, at which they were kept for 1 h. The tubes were left overnight in the oven to cool down to room temperature.

The CO_2_ gas collected was mixed with H_2_ and reduced to solid graphite using Fe as catalyst. The combustion and graphitization process is described by Genberg et al. (2010). The graphite was then pressed into small aluminum holders and inserted to the AMS sample wheel. Each serum sample gave approximately 100–150 μg of carbon. Primary standards (OxI), secondary standards (IAEA-C6 and IAEA-C7) and blanks (made from pre-cleaned fossil carbon-containing materials) were graphitized using the same procedure as that for the serum samples.

The ^14^C analysis of the graphitized serum samples, standards and blanks was performed with the Single Stage Accelerator Mass Spectrometer (SSAMS) at Lund University (Skog. 2007; Skog et al. 2010). The F^14^C values obtained from this analysis were used in the Calibomb program (Reimer et al. 2004) using the North Hemisphere zone 1 calibration data set (Hua and Barbetti 2004). The calibration set was smoothed to 0.5-year lifespan of the samples, and the resolution, which defines the minimum time required to distinguish separate calibration ranges, was chosen to be 0.5 years (http://intcal.qub.ac.uk/CALIBomb/frameset.html: CALIBomb home page, December 2011).

#### δ^13^C and δ^15^N measurements

The weight of each sample was required to be 2.5–3 mg. Thus, 3 μl of serum was transferred to a tin capsule (Säntis Analytical, SA76980502, 3.3 mm × 5 mm) and weighed. Each tin capsule was placed in a numbered well of a 96-well microwell plate, and the samples were dried at 80 °C for 1 h. The tin capsules were then folded and sent to the Environmental Isotope Laboratory (EIl) of the Earth and Environmental Science Division of the University of Waterloo in Ontario, Canada, where the δ^13^C and δ^15^N analysis was performed using a Delta Plus, Continuous Flow IRMS as described by Meier-Augenstein (1999) coupled to a Carlo Erba Elemental Analyzer/CHNS-O EA1108—Italy. According to the sample material type analyzed, the used standards are IAEA-N1 and IAEA-N2 (both ammonium sulfate) for nitrogen and IAEA-CH6 (sugar) and EIL-72 (cellulose), with supplementary international and internal laboratory reference material, for carbon. The corresponding measurement uncertainties were typically ±0.2 ‰ for carbon and ±0.3 ‰ for nitrogen, for clean ball-milled standard material. Depending on the homogeneity, the type and the amount of sample, the uncertainty could rise. This can be overcome through sample repeats. (William Mark, EIL, personal communication, December 2011).

## Results and discussion

**Fig. 2:**
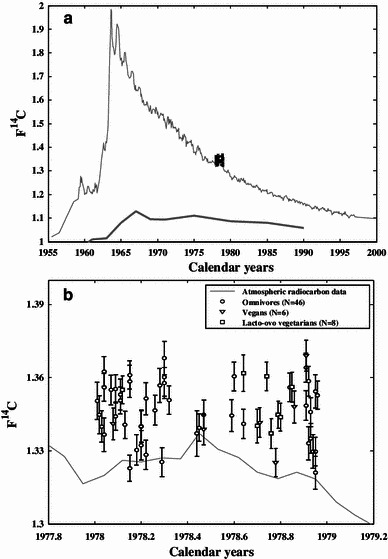
**a** Measured radiocarbon levels in the serum samples analyzed in the present study (*x* points) and the post-bomb radiocarbon data for the atmosphere (thin *gray line*) in the Northern Hemisphere, zone 1, bounded in the south by latitude 40°N (Hua and Barbetti 2004). Marine post-bomb radiocarbon data (*thick gray line*) from the Barents Sea are also presented (Kalish et al. 2001). **b** Measured radiocarbon levels in the serum samples analyzed in the present study, according to diet groups

**Fig. 3:**
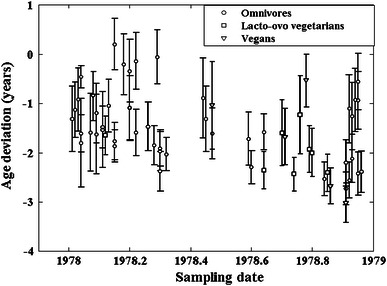
The sampling dates of the serum samples versus the age deviation (difference between Calibomb date and sampling date), in years

The results of the F^14^C, δ^13^C and δ^15^N measurements are presented in Table 2 (for omnivores), Table 3 (for vegans) and Table 4 (for lacto-ovo vegetarians). The F^14^C values of the secondary standards were all within 1.3 SD of the consensus values. Analysis of duplicate samples indicated that the average precision (1 SD) in δ^13^C was 0.06 ‰ and in δ^15^N 0.19 ‰. The majority of the F^14^C values obtained from the serum samples exceeded the atmospheric F^14^C values of the corresponding months of 1978 to various degrees, as can be seen in Fig. 2a, b. Figure 2b shows the results for the various types of diet. The dates given by the Calibomb software ranged from 1975.9 to 1978.4, and the deviation ±1 SD between Calibomb date and sampling date varied between −3.0 ± 0.4 and 0.2 ± 0.5 year (see Fig. 3). The average age deviation was −1.5 ± 0.7 years.

**Table 2:** Values of δ^13^C, δ^15^N and F^14^C determined from the serum samples of omnivores

Sample Id	Group (YoB-sex)	Sampling date	δ^13^C (‰)	δ^15^N (‰)	F^14^C	F^14^C uncertainty [1 standard deviation (SD)]	Calibomb date	Calibomb uncertainty (years)	Age deviation (years)	Age deviation uncertainty (years)
Geo930	1931-F	1,978.92	−22.33	10.85	1.3331	0.0068	1,977.81	0.69	−1.11	0.69
Geo931	1931-F	1,978.93	−22.03	10.64	1.3459	0.0059	1,976.81	0.49	−2.12	0.49
Geo932	1931-F	1,978.92	−22.83	9.92	1.3586	0.0061	1,976.36	0.36	−2.56	0.36
Geo933	1931-F	1,978.93	−22.40	10.68	1.3351	0.0061	1,977.68	0.68	−1.26	0.68
Geo934	1931-F	1,978.94	−21.74	11.17	1.3297	0.0069	1,978.01	0.64	−0.93	0.64
Geo935	1931-F	1,978.95	−22.78	10.19	1.3544	0.0069	1,976.52	0.44	−2.43	0.44
Geo936	1931-F	1,978.95	−22.54	10.24	1.3297	0.0058	1,978.01	0.59	−0.94	0.59
Geo937	1931-F	1,978.96	−22.62	10.15	1.3528	0.0058	1,976.57	0.42	−2.39	0.42
Geo938	1931-F	1,978.91	−22.80	10.10	1.3486	0.0061	1,976.71	0.47	−2.21	0.47
Geo939	1931-F	1,978.95	−22.78	9.94	1.3210	0.0068	1,978.39	0.57	−0.56	0.57
Geo940	1931-M	1,978.60	−22.64	10.26	1.3605	0.0060	1,976.30	0.34	−2.30	0.34
Geo941	1931-M	1,978.84	−22.58	10.75	1.3560	0.0059	1,976.30	0.34	−2.54	0.34
Geo942	1931-M	1,978.44	−22.44	9.74	1.3371	0.0093	1,977.55	0.81	−0.89	0.81
Geo943	1931-M	1,978.91	−22.85	10.60	1.3641	0.0059	1,976.18	0.33	−2.73	0.33
Geo950	1938-F	1,978.03	−22.31	10.01	1.3400	0.0064	1,977.11	0.64	−0.92	0.64
Geo951	1938-F	1,978.20	−22.40	10.11	1.3401	0.0067	1,977.10	0.66	−1.10	0.66
Geo952	1938-F	1,978.20	−22.40	10.75	1.3400	0.0065	1,977.11	0.65	−1.09	0.65
Geo953	1938-F	1,978.20	−22.03	11.13	1.3323	0.0060	1,977.86	0.64	−0.34	0.64
Geo954	1938-F	1,978.18	−21.93	10.79	1.3304	0.0062	1,977.97	0.62	−0.21	0.62
Geo955	1938-F	1,978.22	−21.53	10.87	1.3284	0.0060	1,978.08	0.58	−0.14	0.58
Geo956	1938-F	1,978.30	−22.59	10.51	1.3681	0.0068	1,975.92	0.40	−2.38	0.40
Geo957	1938-F	1,978.30	−22.40	10.62	1.3578	0.0063	1,976.39	0.37	−1.91	0.37
Geo958	1938-F	1,978.30	−22.61	10.28	1.3602	0.0078	1,976.34	0.40	−1.97	0.40
Geo959	1938-F	1,978.32	−22.35	11.11	1.3508	0.0059	1,976.29	0.34	−2.03	0.34
Geo960	1938-M	1,978.04	−22.06	10.67	1.3560	0.0057	1,976.44	0.38	−1.61	0.38
Geo961	1938-M	1,978.11	−22.65	10.59	1.3506	0.0055	1,976.63	0.42	−1.48	0.42
Geo962	1938-M	1,978.15	−22.10	10.65	1.3585	0.0066	1,976.38	0.38	−1.77	0.38
Geo963	1938-M	1,978.15	−22.37	10.40	1.3611	0.0058	1,976.28	0.33	−1.87	0.33
Geo964	1938-M	1,978.15	−22.28	10.13	1.3228	0.0054	1,978.35	0.52	0.20	0.52
Geo965	1938-M	1,978.22	−22.57	10.17	1.3514	0.0066	1,976.63	0.47	−1.59	0.47
Geo966	1938-M	1,978.26	−22.72	9.93	1.3466	0.0061	1,976.79	0.49	−1.47	0.49
Geo967	1938-M	1,978.29	−22.03	10.21	1.3254	0.0059	1,978.23	0.55	−0.06	0.55
Geo970	1949-F	1,978.28	−22.22	10.20	1.3569	0.0070	1,976.43	0.40	−1.85	0.40
Geo971	1949-F	1,978.47	−22.33	10.32	1.3448	0.0060	1,976.86	0.51	−1.61	0.51
Geo972	1949-F	1,978.64	−22.07	10.13	1.3411	0.0060	1,977.07	0.36	−1.58	0.36
Geo973	1949-F	1,978.59	−22.44	10.75	1.3445	0.0065	1,976.87	0.54	−1.72	0.54
Geo980	1949-M	1,978.01	−22.69	10.36	1.3505	0.0077	1,976.70	0.52	−1.32	0.66
Geo981	1949-M	1,978.02	−22.50	10.32	1.3448	0.0079	1,976.90	0.61	−1.13	0.56
Geo982	1949-M	1,978.04	−22.08	10.23	1.3366	0.0069	1,977.58	0.75	−0.46	0.23
Geo983	1949-M	1,978.04	−21.90	10.96	1.3626	0.0061	1,976.24	0.33	−1.80	0.90
Geo984	1949-M	1,978.07	−21.94	10.33	1.3550	0.0061	1,976.49	0.41	−1.59	0.79
Geo985	1949-M	1,978.09	−22.44	10.67	1.3442	0.0058	1,976.89	0.51	−1.20	0.60
Geo986	1949-M	1,978.09	−22.60	9.86	1.3554	0.0058	1,976.47	0.38	−1.63	0.81
Geo987	1949-M	1,978.11	−21.85	10.59	1.3531	0.0064	1,976.57	0.45	−1.54	0.77
Geo988	1949-M	1,978.13	−22.24	10.45	1.3407	0.0056	1,977.08	0.57	−1.05	0.53
Geo989	1949-M	1,978.45	−22.44	10.10	1.3395	0.0056	1,977.13	0.60	−1.32	0.66
Average			−22.36	10.42	1.35		1,976.96		−1.45	0.53
1 SD			0.31	0.36	0.01		0.67		0.71	0.16

The age deviation is the difference between the Calibomb date and the sampling date. Uncertainties are represented by 1 SD

*YoB* year of birth, *F* female, *M* male

**Table 3:** Values of δ^13^C, δ^15^N and F^14^C obtained from the serum samples from vegans

Sample Id	Group (YoB-sex)	Sampling date	δ^13^C (‰)	δ^15^N (‰)	F^14^C	F^14^C uncertainty [1 standard deviation (SD)]	Calibomb date	Calibomb uncertainty (years)	Age deviation (years)	Age deviation uncertainty (years)
Geo944	1931-M	1,978.86	−22.45	9.92	1.3480	0.0066	1,976.18	0.36	−2.68	0.36
Geo945	1931-M	1,978.91	−22.71	7.85	1.3690	0.0063	1,975.89	0.40	−3.02	0.40
Geo946	1931-M	1,978.78	−22.71	9.07	1.3252	0.0058	1,978.24	0.54	−0.54	0.54
Geo968	1938-M	1,978.08	−22.33	10.03	1.3410	0.0049	1,977.24	0.49	−0.85	0.50
Geo974	1949-F	1,978.71	−21.71	9.85	1.3416	0.0061	1,977.03	0.57	−1.68	0.57
Geo975	1949-F	1,978.47	−22.17	9.64	1.3387	0.0063	1,977.44	0.89	−1.04	0.89
Average			−22.35	9.39	1.34		1,977.00		−1.64	0.54
1 SD			0.38	0.83	0.01		0.86		1.02	0.19

The age deviation is the difference between the Calibomb date and the sampling date. Uncertainties are represented by 1 SD

*YoB* year of birth, *F* female, *M* male

**Table 4:** Values of δ^13^C, δ^15^N and F^14^C from the serum samples for lacto-ovo vegetarians

Sample Id	Group (YoB-sex)	Sampling date	δ^13^C (‰)	δ^15^N (‰)	F^14^C	F^14^C uncertainty [1 standard deviation (SD)]	Calibomb date	Calibomb uncertainty (years)	Age deviation (years)	Age deviation uncertainty (years)
Geo947	1931-M	1,978.74	−22.31	9.30	1.3606	0.0059	1,976.30	0.34	−2.44	0.34
Geo948	1931-M	1,978.79	−21.98	9.85	1.3448	0.0055	1,976.87	0.48	−1.93	0.48
Geo949	1931-M	1,978.85	−23.10	9.18	1.3561	0.0061	1,976.45	0.38	−2.41	0.38
Geo969	1938-M	1,978.12	−22.41	9.98	1.3551	0.0057	1,976.48	0.39	−1.65	0.39
Geo976	1949-F	1,978.64	−23.49	9.64	1.3619	0.0075	1,976.29	0.38	−2.36	0.38
Geo977	1949-F	1,978.80	−23.10	8.48	1.3438	0.0057	1,976.81	0.49	−2.00	0.49
Geo978	1949-F	1,978.70	−23.23	9.62	1.3402	0.0069	1,977.10	0.67	−1.60	0.67
Geo979	1949-F	1,978.76	−22.84	9.68	1.3371	0.0061	1,977.54	0.79	−1.23	0.79
Average			−22.81	9.47	1.35		1,976.77		−1.95	0.49
1 SD			0.52	0.48	0.01		0.47		0.44	0.16

The age deviation is the difference between the Calibomb date and the sampling date. Uncertainties are represented by 1 SD

*YoB* year of birth, *F* female, *M* male

As mentioned in the introduction, several factors may cause a difference between the date from atmospheric ^14^CO_2_ data and the ^14^C bomb-pulse date in human serum. It is difficult to estimate whether ^14^C contamination from nuclear installations (increasing the specific activity of ^14^C in foodstuffs) or fossil fuel emissions (lowering the specific activity ^14^C in foodstuffs) has affected the samples. In any case, both these effects would probably be overshadowed by other (more dominating) factors. Since the majority of the measured F^14^C values exceeded the corresponding atmospheric ^14^CO_2_ data (none, within the uncertainty of the measurements, is below the atmospheric F^14^C data), the delay between the production and consumption of the foodstuffs is probably the dominating factor.

Georgiadou and Stenström (2010) concluded that the marine component of the diet has the potential to lower the average F^14^C in the human diets to varying degrees, which can produce an age deviation in bomb-pulse dating. There are few radiocarbon data sets from the marine environment. Apart from atmospheric ^14^C data, Fig. 2a also shows post-bomb marine ^14^C data obtained from otoliths and Arcto-Norwegian cod collected in the Barents Sea (Kalish et al. 2001). Barent Sea can be regarded as *clean,* meaning not radiocarbon-contaminated by human sources, sea water in the Northern hemisphere. This figure clearly illustrates the difference between ^14^C concentration in the marine and atmospheric reservoirs and provides a rough estimation of the radiocarbon concentration of marine food consumed by the human subjects included in the present study. However, the influence of the marine component of the diet is obviously smaller than other effects, such as the effect of the delay time; otherwise, the serum F^14^C values would have been lower than the atmospheric ^14^CO_2_ values.

**Fig. 4:**
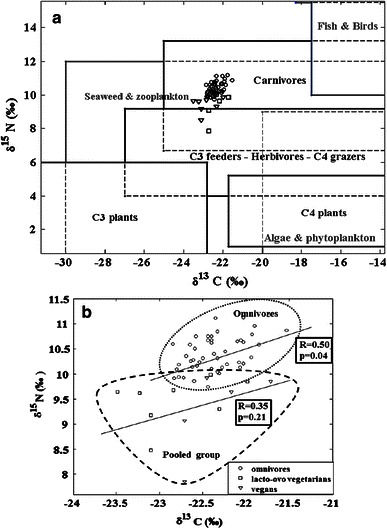
**a** The δ^13^C and δ^15^N values from the serum samples analyzed in the present study included in the isotopic trophic level diagram. **b** Grouped samples from omnivore subjects and from lacto-ovo vegetarian and vegan subjects (pooled group) and regression lines in a δ^13^C versus δ^15^N diagram

In order to further understand and explain the observed F^14^C excess in the serum samples and to estimate the marine component of the diet, the δ^13^C and δ^15^N values for each sample were inserted into the generalized isotopic trophic level diagram of Fig. 1 (Fig. 4a). It should be noted that the differences in δ^13^C and δ^15^N between human tissue and human diet, mentioned above (Schoeller et al. 1986), have not been considered in this diagram. According to the conclusions presented by Schoeller et al. 1986, this should be indicative of even lower values for their respective diets. The data are plotted in more detail in Fig. 4b, where it can clearly be seen that the majority of the δ^15^N values of the vegans and the lacto-ovo vegetarians (7.85–10.03 ‰) are lower than those of the omnivores (9.7–11.2 ‰). There is also an indication that the omnivores do not exhibit as low δ^13^C values as some of the lacto-ovo vegetarians, which is consistent with the dietary trend in the generalized isotopic trophic level diagram in Fig. 1. From Fig. 4b, it is clear that, even though the pooled group consisting of lacto-ovo vegetarians and vegans is not completely separated from the omnivores, δ^13^C and δ^15^N add valuable information on probable dietary habits.

The correlation between δ^13^C and δ^15^N is statistically significant (*R* = 0.50, *p* = 0.04) for omnivore subjects (see Fig. 4b), which suggests that the diet of these subjects is dominated by plants from the C3 group, rather than the C4 group (see Fig. 1). The correlation between δ^13^C and δ^15^N is not statistically significant for vegan and lacto-ovo vegetarians (*R* = 0.35, *p* = 0.21) (Fig. 4b). This may be a result of the absence of fish and meat products in their diets.

**Fig. 5:**
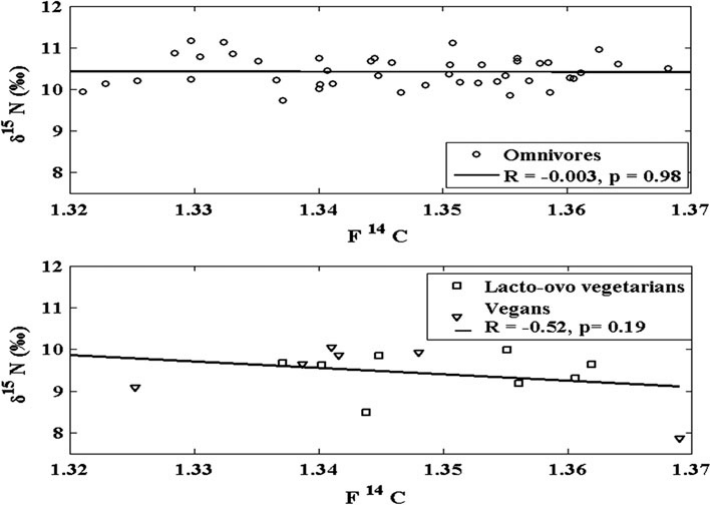
F^14^C versus δ^15^N values for each subject with known dietary habit

Figure 5 shows the F^14^C values of the samples versus their respective δ^15^N values. It can be seen from this figure that δ^15^N has a little variation precluding to diet and that the δ^15^N values in the samples from lacto-ovo vegetarians and vegans are concentrated in the lower regions (8.5–10 and 7.9–10 ‰, respectively, compared to 9.7–11 ‰ for omnivores). According to the generalized isotopic trophic level diagram (Fig. 1), high δ^15^N values ought to correspond to a higher consumption of fish products than low δ^15^N values. If consumption of fish should be visible in the ^14^C data, the high δ^15^N values of fish should have a lowering effect on the F^14^C values (since the marine F^14^C is lower than the terrestrial F^14^C, see Fig. 2). The correlation between F^14^C and δ^15^N is, however, not significant for any type of diet (*R* = −0.003, *p* = 0.98 for omnivores and *R* = −0.52, *p* = 0.19 for lacto-ovo vegetarians and vegans). Thus, for the samples of this study, δ^15^N values do not seem useful for age correction purposes of bomb-pulse dating.

**Fig. 6:**
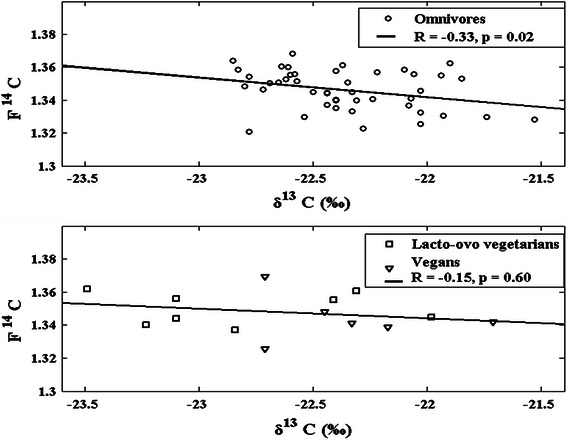
δ^13^C versus F^14^C values for each subject with known dietary habit

There is, however, a significant negative correlation (*R* = −0.33, *p* = 0.02, see Fig. 6) between F^14^C and δ^13^C for omnivores. In the generalized isotopic trophic level diagram, it is reasonable to assume that the subjects of the current study (inhabitants of the city of Malmö) consume considerably more vegetarian food of the C3 than the C4 group. Thus, high δ^13^C values possibly correspond to a higher fraction of marine diet than low δ^13^C values. Keeping the lower F^14^C of marine diet compared to terrestrial diet in mind, the negative correlation between F^14^C and δ^13^C presented in Fig. 6 makes sense. For the *pooled* group of vegans and lacto-ovo vegetarians, there is no significant correlation between F^14^C and δ^13^C (*R* = −0.15, *p* = 0.60, see Fig. 6), which might be interpreted as the actual lack of fish in their diets.

**Fig. 7:**
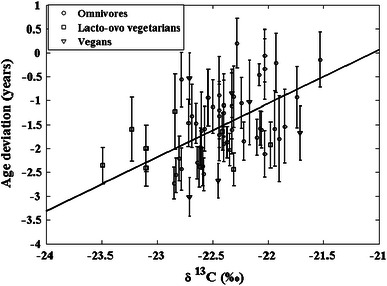
δ^13^C versus the age deviation (difference between Calibomb date and sampling date), for all the samples of this study

The observation of a linear negative correlation between F^14^C and δ^13^C for omnivores is inspired to Fig. 7, which shows the age deviation, that is, the difference between the Calibomb date and the sampling date, as a function of δ^13^C for all the samples of this study. Although rather scattered, the data are strongly linearly correlated (*R* = 0.51, *p* < 0.0001). It has to be noted that, based on the dietary habits of the subjects, only the omnivores show a strong linear correlation of the δ^13^C values with the age deviation (*R* = 0.62, *p* < 0.0001), while the δ^13^C values from samples of the pooled group alone do not correlate with the age deviation (*R* = 0.19, *p* = 0.49). No definite conclusion can though be drawn, since the number of the subjects of the pooled group was quite limited. The fact that the age deviation is less for omnivore samples with rather high δ^13^C values can be interpreted as a result from intake of fish products (high δ^13^C values, see Fig. 1). From Fig. 2a, it is apparent that consumption of food represented by the marine calibration curve has the effect of giving dates that are younger. This is valid even if the marine food is stored before consumption. Terrestrially produced food in the diet, on the other hand, is older than the sampling date, leading to older dates in the calibration. Thus, it seems like the marine foodstuffs neutralize the delay time caused by terrestrial foodstuffs, an effect that increases with increasing intake of marine food, that is with increasing δ^13^C values.

**Fig. 8:**
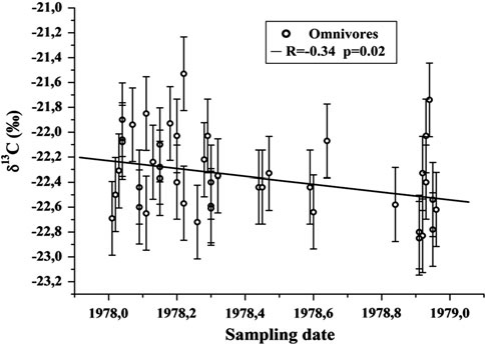
Sampling date versus δ^13^C for the omnivore diet group

Medium negative correlation was found between the sampling date and the δ^13^C values of the samples from omnivore subjects (Fig. 8). This leads to the speculation of a diet change toward more consumption of C3 plants, or less marine food consumption, over the year. No such correlation was found for samples of the pooled group, possibly due to the lack of meat and fish in their diet as well as the limited consumption of C4 plants.

**Fig. 9:**
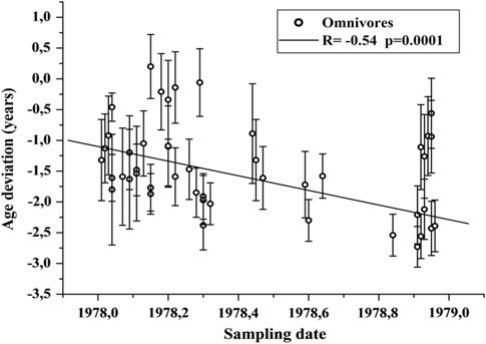
Sampling date versus age deviation for the omnivore diet group

For the omnivore subjects, a strong correlation was found between sampling date and age deviation (Fig. 9). Speculatively, this might be caused by higher portion of stored food at the end of the year.

**Fig. 10:**
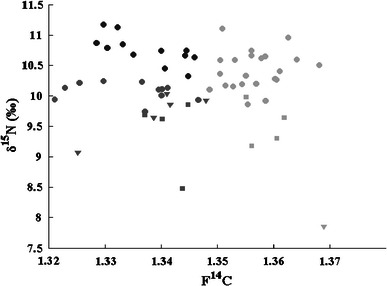
The three cluster groups, in a F^14^C versus δ^15^N diagram

**Fig. 11:**
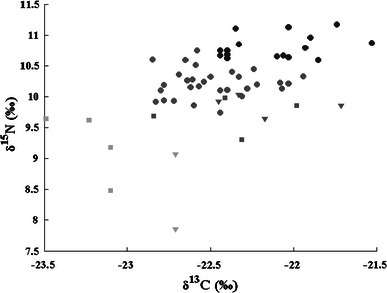
The three cluster groups in a δ^13^C versus δ^15^N diagram

Cluster analysis was applied to the data for identifying sub-groups of homogeneous similar elements. The cluster analysis technique is an exploratory data analysis tool that identifies a non-specified number of data subsets depending on the similarities and differences between elements (Wilks 2006). The data were pre-filtered by principal component analysis (Jackson 1991) prior of use to avoid the interference of noise in the analysis. The cluster analysis leads to reasonable results, when it was based on the δ^13^C and δ^15^N values of the samples. The results of the clustering (δ^15^N vs. F^14^C and δ^15^N vs. δ^13^C) are presented in Figs. 10 and 11. In both cases, the cluster analysis divided the data in three sub-groups containing similar elements. From Fig. 10, it can be seen that all the samples from vegan subjects (except for one) are in the same group (shown in dark gray), which presents lower values of both δ^15^N and F^14^C. Some of the values from the omnivores and about 50 % of the values from the lacto-ovo vegetarians are found in this group. It is expected that the δ^15^N values will be quite low in the majority of the samples from vegans, since they do not eat fish or meat which have the highest values of δ^15^N according to the isotopic trophic level diagram in Fig. 1. The group denoted in lighter gray in Fig. 10 is characterized by higher levels of F^14^C and is populated by values from both omnivores and lacto-ovo vegetarians together with one vegan. The relatively high F^14^C value of this single vegan could be attributed to a possible high consumption of stored food (e.g. canned or frozen).

The darkest labeled group shown in Fig. 10, characterized by high δ^15^N and low F^14^C, is composed of the results originating only from omnivores. Bearing in mind the isotopic trophic level diagram, it could be speculated that these omnivores ate particularly a lot of fish and meat.

Regarding the results for δ^13^C versus δ^15^N, (Fig. 11), a cluster of values obtained from omnivores, with higher δ^13^C (−22.2 ‰) and δ^15^N (10.7 ‰) averages compared to the respective averages of all the omnivore samples of this study together (−22.46 and 10.4 ‰, respectively), can be once more observed (darkest labeled group). Considering the fact that this group has an F^14^C average value (1.34) which is lower than the respective average of the total omnivore samples (1.35), this could be a hint that the samples of the darkest marked group, all coming from omnivore subjects, are possibly separated from the rest of the omnivore samples, due to excessive fish consumption of the subjects that they are coming from. The light-grayed marked group in this figure includes no sample from the omnivore subjects and has the lower average δ^13^C and δ^15^N values. This is expected since the subjects of this group do not eat meat and fish. It should be noted that these two food groups have higher δ^13^C and δ^15^N averages compared to the other foodstuff groups, according to Fig. 1.

The cluster analysis did not reveal any dependence of δ^13^C, δ^15^N or F^14^C on sex or age of the subjects, which indicates no difference in dietary habits between sexes or the various age groups.

Finally, cluster analysis of δ^13^C versus F^14^C did not reveal any additional information.

## Conclusion

One aim of the present study was to assess the magnitude of inter-individual variations in human ^14^C data depending on dietary habits, in order to estimate the precision and accuracy of bomb-pulse dating in human tissues. The possible effects of dietary habits (omnivore, lacto-ovo vegetarian or vegan), age and sex were also studied, as well as whether δ^13^C and δ^15^N analysis using IRMS can provide additional information on the diet.

The measurements of human blood serum samples taken in 1978 showed an excess in radiocarbon compared with the atmospheric radiocarbon content of the same year, thereby producing dates older than the sampling date. The age difference (±1 SD) between the date estimated from clean-air data and the sampling date varied between −3.0 ± 0.4 and 0.2 ± 0.5 years, with an average age deviation of −1.5 ± 0.7 years. The sampling date appeared to have an effect on the δ^13^C values as well as on the observed age deviation obtained from the ^14^C measurements. This might indicate seasonal changes in dietary habits. No influence of sex or year of birth was found on the radiocarbon content or on the δ^13^C and δ^15^N values.

Stable isotope analysis showed a linear correlation between δ^13^C and δ^15^N for omnivores, but not for the limited number of vegans or lacto-ovo vegetarians in this study. The general trend was that vegans and lacto-ovo vegetarians displayed lower values of δ^13^C and δ^15^N than the omnivores. This corresponds well with the expected δ^13^C and δ^15^N values for the different diet groups and demonstrates the potential of stable isotope analysis as an additional tool in revealing dietary habits in bomb-pulse studies of human material.

Furthermore, cluster analysis revealed that omnivore samples with higher δ^15^N and δ^13^C values and low F^14^C values constitute a separate group. It can be speculated that these samples originate from omnivores consuming a relatively high fraction of marine food and meat compared to the omnivore subjects of the other group. These foodstuffs, especially those of marine origin, have high δ^15^N and δ^13^C values. Marine food also has a lower F^14^C content compared to terrestrial food, which has F^14^C values closer to the atmospheric level. Ultimately, as far as the population of this study is concerned, subjects are expected to consume more C3 (lower δ^13^C) than C4 plants (higher δ^13^C), both groups with similar δ^15^N and F^14^C levels. This information could indicate that the high δ^13^C and δ^15^N paired diet values are mainly due to food consumption from the food groups comprising fish and birds.

Linear correlation studies showed a statistically significant negative correlation between F^14^C and δ^13^C for the omnivore subjects of this study, but not between F^14^C and δ^15^N. Translated into age deviation, the omnivore subjects displayed less age deviation with increasing δ^13^C values (i.e., with increasing fraction of marine food). An interpretation is that, when using atmospheric data as a calibration data set, marine foodstuffs can give younger dates, while terrestrial food can produce older dates because of the time delay between production and consumption of the food. These two types of dietary component therefore have opposite effects on the Calibomb date obtained. Whether δ^13^C can be used as a general tool to correct the Calibomb dates of human samples, for example, with different sample material or material from subjects with different dietary habits at different times during the bomb pulse, needs to be further investigated. However, it is evident that δ^15^N and δ^13^C analysis can be a valuable tool in combination with bomb-pulse dating to spot samples where extreme dietary conditions might influence the radiocarbon date obtained from the atmospheric calibration data set used in the Calibomb calibration program. In particular, high fractions of terrestrial food may produce dates that are too old, while very high fractions of marine foodstuffs in the diet may produce Calibomb dates that are too young. However, the latter was not observed for the Swedish subjects in the current study.

In conclusion, the dominating factor for the age deviation found by ^14^C bomb-pulse dating for the subjects in this study was probably time delay between production and consumption of the food.

It should be noted that all these conclusions refer only to the particular samples of the study, collected in 1978.

## Footnotes

1 δ^13^C for C_3_ = −26.5 and C_4_ = −12.5 ‰ (Chisholm 1989 and references therein). δ^13^C for C_3_ = −26 and C_4_ = −12 ‰ (O’Connell 1996 and references therein).
